# Changes in Metallothionein Levels in Freshwater Mussels Exposed to Urban Wastewaters: Effects from Exposure to Heavy Metals?

**Published:** 2007-03-29

**Authors:** F. Gagné, C. Gagnon, P. Turcotte, C. Blaise

**Affiliations:** St. Lawrence Centre, Environment Canada, 105 McGill, Montréal, Québec, Canada H2Y 2E7

**Keywords:** Metallothioneins, bivalves, Municipal effluents, inflammation, metal bioavailability

## Abstract

Municipal effluents are complex mixtures of compounds such as heavy metals, aromatic and aliphatic hydrocarbons, and micro-organisms and are released in aquatic ecosystems. The purpose of this study was to verify whether changes in metallothioneins (MT) were associated with the accumulation of labile metals in tissue of freshwater mussels exposed to the dispersion plume of a major municipal effluent. Mussels were placed in experimental cages deployed at sites 1.5 km upstream, 8 km downstream and 12 km downstream of the outfall of a major, primary-treated municipal effluent in the St. Lawrence River (Québec, Canada). Mussels were analysed for MT and labile zinc levels in their gonads, gills and digestive glands. Lipogenic enzyme (isocitrate and glucose-6-phosphate dehydrogenase) and arachidonic acid cyclooxygenase (COX) activities were also measured in gonad and gill tissues. Although MT was induced in all the tissues examined, the results showed that labile zinc levels were significantly reduced in gill and gonad tissues, with an increase observed only at the 12 km downstream site in the digestive gland. COX activity was readily induced in gills and gonads. Glucose-6-phosphate dehydrogenase activity was reduced at both downstream sites, but isocitrate dehydrogenase activity was significantly induced at the farthest (12 km) site. Analysis of covariance revealed that MT levels in gills were more influenced by COX activity than with distance in the dispersion plume and was negatively correlated with labile zinc levels. In conclusion, MT induction was inversely related to the levels of labile zinc but positively so with the inflammation biomarker COX. Hence, the induction of MT in mussels exposed to the municipal effluent of a large city appears to be associated with either inflammatory processes or as compensation for the loss of labile essential metals. We propose that the simple and complimentary parameters of labile zinc and COX evaluations be used to link MT induction with divalent heavy metal exposure in environmental studies dealing with various type of contaminants in such complex contaminant mixture effluents.

## Introduction

Municipal effluents are recognized as major sources of pollution for aquatic ecosystems. These anthropogenic sources of pollution release significant amounts of contaminants such as micro-organisms, heavy metals, pesticides, estrogens, and aliphatic/polyaromatic hydrocarbons (PAHs) that can potentially harm aquatic biota (Chambers et al. 1997). Moreover, municipal effluents are rich in organic matter (Imai et al. 2002) that can modulate the availability of organic and inorganic compounds like heavy metals through sequestration by low- and high-molecular-weight complexes (Pan and Wang, 2004; Gagnon et al. 2006). Freshwater mussels are particularly at risk from this type of pollution because they are sessile and filter large amounts of surface water, including suspended particles and colloids. Municipal effluents were shown to contain heavy metals and have the potential to induce toxicity in exposed organisms (Pessala et al. 2004).

Metallothioneins (MT) are low-molecular-weight proteins containing 25 to 30% cysteine that sequester and limit the toxicity of divalent heavy metals in cells such as zinc, copper, cadmium, mercury and nickel (Cosson, 2000). The induction of MT in tissues and the levels of metals bound to the protein consist the most common ways to assess the early biological effects or defence mechanisms against potentially toxic levels of metals in aquatic organisms (Amiard et al. 2006). Metals bound to MT are considered less labile and thus less toxic, but the exact mechanism of protection against metals is complex. Indeed, the mechanism of action of MT depends on the degree of heavy metal exposure of tissues and the MT response is not the only physiological mechanism at play for heavy-metal sequestration in bivalves (Bonneris et al. 2005). Under chronic exposure, cadmium, copper and zinc were principally located in calcium concretions in the gills and acting as non-inducible metal sinks in unionids. Moreover, metal concentrations increased proportionately in both the concretions and the MT pool along a polymetallic gradient, suggesting equilibrium between these two compartments. At low levels of exposure, MT was shown to maintain zinc and copper homeostasis in trout hepatocytes, suggesting that MT sequesters essential metals to maintain the cell’s biogenic metal homeostatis (Gagné et al. 1990). At high levels of exposure (i.e. approaching toxicity and lethality), MT was shown to sequester toxic metals from high-molecular-weight proteins and MT levels were significantly correlated with the metal concentration in exposed tissues or in the cytosol fraction (Couillard et al. 1995; Dragun et al. 2004).

However, the MT response was not always specific to divalent heavy metal exposure. Indeed, MT was induced by alkylating agents (Robson et al. 1994), cortisol (Hyllner et al. 1989), inflammation or infections (Coyle et al. 1995; Regala and Rice, 2004), and oxidative stress (Bauman et al. 1991). The production of nitric oxide (a precursor of peroxinitrite, a strong oxidizing agent) during infections was shown to induce MT by displacing zinc from the protein in macrophages (Katakai et al. 2001). Infection by a digenean parasite was shown to have a small influence on MT expression perhaps through a similar inflammatory pathway (Baudrimont et al. 2006). The absence of hydrophobic regions of the MT protein makes metal sequestration particularly resilient to change during thermal stress; indeed, MT was considered a heat-stable form of divalent metal. The increase in intracellular labile zinc by xenobiotic metals was proposed as a means for evaluating heavy metal exposure more specifically (Gagné and Blaise, 1996). Although the MT biomarker has been used successfully in environments contaminated by heavy metal pollution gradients (Couillard et al. 1995a; Giguère et al. 2006), studies on MT variations in mixed-type of contamination are lacking. Induction of MT in complex mixtures such as urban wastewaters was observed in a previous study in freshwater mussels exposed to the dispersion plume but with a concomitant decrease in metal bioaccumulation in the corresponding tissues even though the effluent contained metals (Gagnon et al. 2006). MT levels are expected to follow metal bioaccumulation patterns in tissues, such that the remobilization of metals from high-molecular-weight proteins to MT supposedly to reduce the toxic impacts of metals (Perceval et al. 2004; Couillard et al. 1995b). This raises the possibility that heavy metal exposure was not the principal etiological factor in MT expression in freshwater mussels exposed to complex mixtures like municipal effluents. In the present study, inflammation was assessed by following cyclcooxygenase (COX) activity which is the rate limiting enzyme in the production of prostaglandins from arachidonic acid (Fujimoto et al. 2002). Prostaglandins are also produced during the spawning process in bivalves (Osada and Nomura, 1990) indicating that biomarker should not be used in spawning mussels.

The objective of this study was to re-examine the MT response in freshwater mussels exposed *in situ* to urban effluents of a large city. Confirmation of exposure to the effluent plume was achieved by following the activity of lipogenic enzymes that are induced to assit steroidogenesis (Mori et al. 1967). MT levels in three tissues (gills, digestive gland and gonad) were examined in relation to labile zinc levels, COX activity and reported metal bioaccumulation in mussel tissues. The measurement of labile zinc and MT levels in organisms exposed to complex mixtures was proposed to confirm the aetiology of heavy metal exposure for MT in bivalves.

## Methods

### Mussel collection and cage preparation

**Figure 1:**
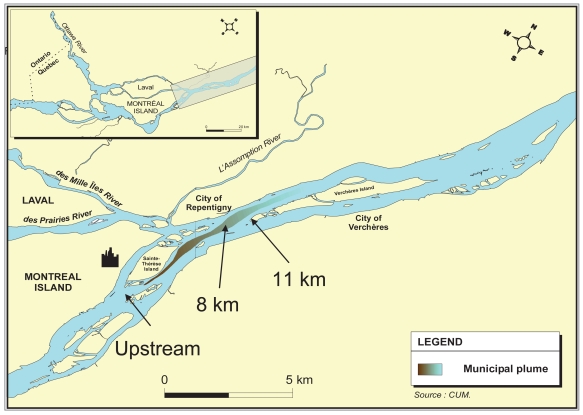
Map of the study area showing the location of mussel cages along the effluent dispersion plume.

Mussels were collected in lake de L’Achigan in the Laurentians, Quebec, Canada, in an area known to abound in *Elliptio complanata* (Downing and Downing, 1992) during the first week of May 1999 (water temperature 6–8°C). They were maintained in tanks using dechlorinated, UV-treated and aerated tap water at 15°C for several weeks and fed three times a week with *Selenastrum capricornutum* micro-algae or cultures of commercial coral reef solution. Experimental cages of *E. complanata* mussels were prepared according to Applied Biomonitoring’s methodology (Salazar and Salazar, 2001). Briefly, 12–15 *Elliptio* mussels of similar sizes (7–8 cm) were placed in each of four nets. Each net was then attached to a PVC frame (1 m^2^ surface area), tied to a 20-kg cinder block and marked with a buoy. Four cages were deployed at each of three sites located 1.5 km upstream and 8 km and 12 km downstream of a municipal effluent outfall (Figure 1). They were submerged in the St. Lawrence River for 90 days, from the beginning of June until the beginning of September 2001.

### Biomarker analyses

At the end of the exposure period, the cages were taken up and immediately brought to the laboratory for determination of shell length, shell weight, and soft tissue weight. The tissues were homogenized in ice-cold 25 mM Tris-acetate, pH 7.5, containing 100 mM NaCl and 1 mM dithiothreitol with a Teflon pestle tissue grinder (10 passes). Homogenates were centrifuged at 12 000 × *g* and the supernatant (S12) stored at −85°C until biomarker analyses could be performed. Metallothionein (MT) levels were determined in the digestive gland and gills of *E. complanata*. Relative levels of MT-like proteins were determined by the silver saturation assay (Scheuhammer and Cherian, 1986). Briefly, 50 μL of S12 was mixed with one volume of 20 mg/L silver (Ag^1+^) in 100 mM glycine-NaOH, pH 8.5. After a 10-min incubation time at room temperature, the volume was adjusted to 500 μL with glycine buffer and unbound or excess silver was removed by two successive haemoglobin additions and removed by heat denaturation/centrifugation steps. The levels of Ag remaining in the supernatant were determined by Zeeman graphite furnace atomic absorption spectrophotometry. Blanks did not contain the S12 supernatant and standards of rabbit MT were included. The ratio of 12 moles of Ag per mole of MT was assumed in all cases. The data are expressed as nmoles of MT equivalents per mg protein. Total proteins were determined by the method of Bradford (1976). Levels of labile zinc were measured in the supernatant by the fluorescent probe method (Gagné and Blaise, 1996). A volume of 25 μL was mixed with 125 μL of phosphate buffered saline (140 mM NaCl, 1 mM KH_2_PO_4_ and 10 mM Hepes-NaOH, pH 7.4) and 50 μL of 50 μM of N-(6-methoxy-8-quinolyl)-p-toluenesulfonamide (TSQ) probe (prepared in 10% DMSO; Molecular Probes, U.S.A.) was added. After 5 min, fluorescence was measured at 360 nm excitation and 480 nm emission using a microplate reader (Fluorolite-1000). Cyclooxygenase (COX) activity was measured by the oxidation of 2, 7-dichlorofluorescin in the presence of arachidonate (Fujimoto et al. 2002). Briefly, 50 μL of gonad or gill 15 000 × *g* supernatant was mixed in 200 μL of 50 μM arachidonate, 2 μM dichlorofluorescin and 0.1 μg/mL of horseradish peroxidase in 50 mM Tris-HCl buffer, pH 8, containing 0.05% Tween 20. The reaction mixture was incubated for 0, 10, 20 and 30 min at 30 °C, and fluorescence was measured at 485 nm for excitation and 520 nm for emission (Dynatech, Fluorolite 1000). The data were expressed as the increase in relative fluorescence units/(min × mg proteins). Lipogenic activity was determined in mussel gonadal tissues. The activity of glucose-6-phosphate deshydrogenase (G6PD) and isocitrate deshyhdrogenae (ICD) in the S12 fraction was measured by following the appearance of NAD(P)H in the presence of either 1 mM glucose 6-phosphate (NADPH) or isocitrate (NADH) and 0.2 mM of NAD(P)^+^ in 50 mM Hepes-NaOH buffer, pH 7.4, containing 125 mM NaCl. For ICD, 5 mM MnCl_2_ was added to the incubation buffer. The appearance of NADH was measured by microfluorescence at 365 nm excitation and a 450 nm emission (Fluorolite-1000 microplate reader, Dynatech, U.S.A.). The activity was reported by relative fluorescence units/min/mg proteins.

### Data analysis

Twelve *Elliptio complanata* mussels were collected from each of the upstream and the two downstream sites. Critical differences between upstream and downstream groups were appraised using a one-way analysis of variance and critical differences between the upstream and each of the downstream sites were appraised with the Mann-Whitney U test. The biomarkers were also analysed by the Pearson-moment correlation procedure. Significance was set at a level of *p* < 0.05.

## Results

**Figure 2:**
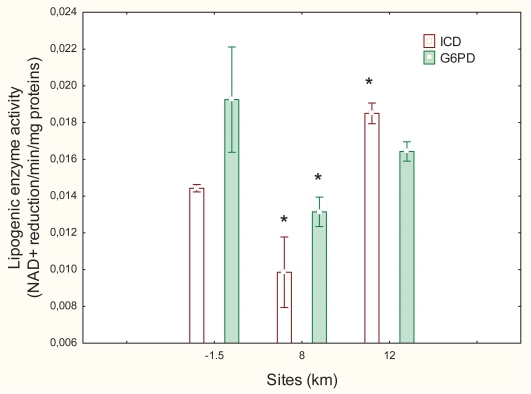
Lipogenic enzyme activity in freshwater mussels exposed to the municipal effluent. The activities of isocitrate deshydrogenase (ICD) and glucose-6-phosphate deshydrogenase were determined in the gonad. The symbol * indicates significance at p < 0.05.

No significant morphological changes were observed in mussels placed 1.5 km upstream, and 8 km and 12 km downstream of the effluent dispersion plume. The activity of lipogenic enzymes was determined in gonad tissues (Figure 2). The activity of ICD was more strongly affected (Anova: F = 15; p < 0.01) than the activity of G6PD (Anova: F = 3.1; p < 0.05). The activity of ICD revealed a more comples pattern of response. ICD activity was significantly induced 1.3-fold at the 12 km downstream site relative to the upstream site but significantly reduced 0.6-fold at the 8 km downstream site. The activity of G6PD was significantly reduced 0.7-fold at the site 8 km downstream of the municipal outfall relative to the upstream site. The G6PD activity between the 8 km and 12 km downstream sites was not statistically different. IDC and G6PD were not significantly correlated.

**Figure 3:**
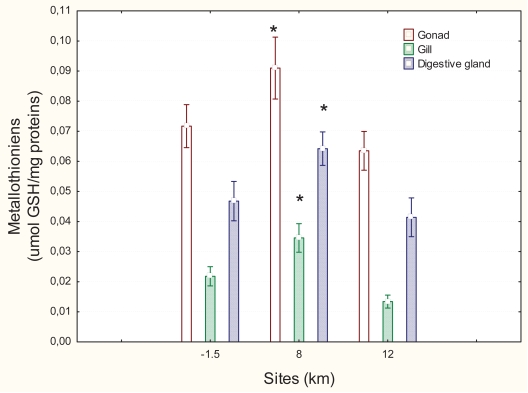
Levels of metallothioneins in mussel tissues. Levels of metallothioneins were determined in mussels that were placed upstream and downstream a municipal effluent source. Significance was set at p < 0.05. *Reprinted from Chemosphere, 62, Gagnon et al. Exposure of caged mussels to metals in a primary-treated municipal wastewater plume, 998–1010. Copyright (2006), with permission from Elsevier.*

The levels of MT were determined in various mussel tissues: gonad, gills and digestive gland (Figure 3). Based on the F-values of ANOVA, MT induction was most significant in gills (F = 11.2; p < 0.01) followed by digestive gland (F = 4.5; p < 0.05) and gonad (F = 3.6; p < 0.05). In gonad, the MT level was marginally increased 1.3-fold (p = 0.07) at the 8 km downstream site and returned to levels comparable to the upstream site at 12 km downstream. In the gill tissues, MT levels rose 1.5-fold at the 8 km downstream site and returned to upstream levels at the 12 km downstream site. In the digestive gland, MT levels rose 1.4-fold at the 8 km downstream site and returned to normal levels at the 12 km downstream site compared to the upstream site. Digestive gland MT was correlated with gonad MT levels (R = 0.39; p < 0.05) and gill MT (R = 0.36; p < 0.05), as shown in Table 1.

**Figure 4:**
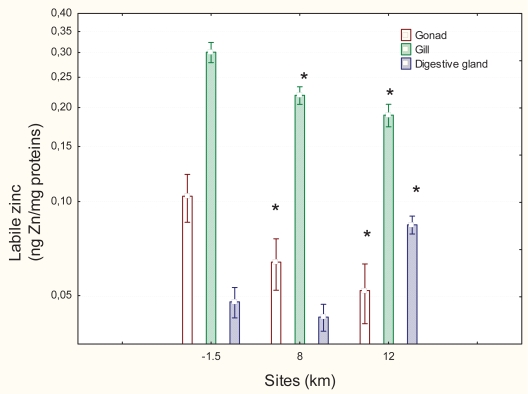
Zinc levels in various mussel tissues exposed to a municipal effluent. Zinc levels were determined in homogenate extract of gonad, gill and digestive gland tissues. Significance was set at p < 0.05.

**Figure 5:**
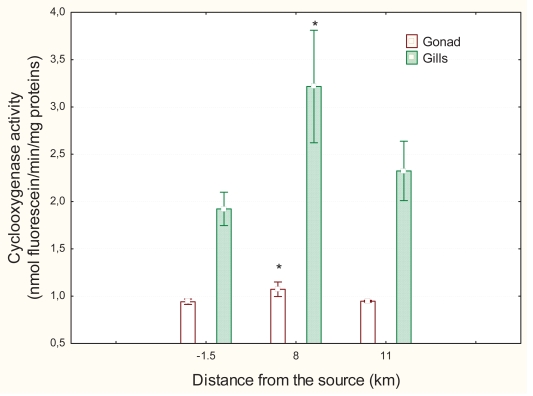
Cyclooxygenase activity in various mussel organs exposed to a municipal effluent. Cyclooxygenase activity was determined in homogenate extract of gonad, gill and digestive gland tissues. Significance was set at p < 0.05.

Levels of labile zinc were measured in the same tissues as for MT to determine whether the expression of MT is due to increased labile zinc pools (Figure 4). The most significant effects were observed in the digestive gland (F = 21; p < 0.001) followed by gills (F = 11; p < 0.01) and gonad tissues (F = 4; p < 0.01). In the digestive gland, Zn levels were significantly increased 1.8-fold at the 12 km downstream site while MT levels returned to upsteam values. Zn levels at the 8 km site did not change in respect to the upstream site but MT levels were significantly induced. However, labile zinc levels in the gills were significantly lower 0.7- and 0.6-fold at the 8 and 12 km downstream sites, respectively, relative to the upstream site. In gonad tissues, the levels of labile zinc were also lower by 0.6- and 0.5-fold at the 8 and 12 km downstream sites, respectively. Zinc levels in gonads were significantly correlated with levels in gills at R = 0.57 p < 0.01 (Table 1). COX activity was determined in gills and gonad tissues (Figure 5). The most significant effects in COX activity were in the gill (Anova F = 4.25; p < 0.05) followed by the gonad (Anova: F = 3.3; p < 0.05). In the gonad, COX activity was increased 1.4-fold at the 8 km downstream site relative to the upstream site. In the gills, COX activity was increased 1.8-fold at the 8 km downstream site compared to the upstream site and 1.3-fold at the 12 km downstream site. COX activity in the gonad and gill tissues was significantly correlated at R = 0.71 (Table 1).

Correlations between the biomarker data were also examined (Table 1). The activity of gonadal ICD for the production of NADH to support lipid synthesis was positively correlated with gonadal COX (R = 0.89) and Zn in digestive gland (R = 0.62). Its activity was negatively correlated with MT in gills (R = −0.64) and COX in gills (R = −0.49). The G6PD activity for the production of NADPH to support lipid synthesis was positively correlated with gonadal Zn (R = 0.54), but was negatively correlated with COX (R = −0.45) and MT in gonad (R = −0.46). Interestingly, the levels of MT were more closely associated with the activity of the inflammation biomarker COX than with the levels of labile zinc. Indeed, gill MT was positively correlated with COX in gills (R = 0.62) and COX in the gonad (R = 0.61) and negatively correlated with Zn in the digestive gland (R = −0.56), with the absence of any correlation with Zn in gills. This observation was further supported by an analysis of covariance of gill MT using either COX or labile zinc as covariates. Indeed, difference in MT levels in gills were not significant between sites when both COX and labile zinc measurements were included as cofactors. The covariate COX had more influence in reducing the F-value (ΔF = 6.6) than did the covariate labile zinc (ΔF = 5.7). However, the major effect of labile zinc in gills was a significant reduction in its levels instead of an increase. Thus, the induction of MT in tissues was not statistically related to the amount of labile zinc in tissues.

## Discussion

Mussels exposed to municipal effluents generally display increased vitellogenin-like proteins, these complex mixtures having been identified as estrogenic to freshwater mussels (Marin and Matozo, 2004; Gagné et al. 2001). In a parallel study at the same sites, freshwater mussels had increased vitellogenin-like proteins in their gonads, confirming that mussels were influenced by the municipal effluent dispersion plume in this large river ecosystem (Gagnon et al. 2006). Furthermore, data on ICD, a lipogenic enzyme implicated in the production of NADH for lipid synthesis, support the presence of estrogenic chemicals at the 12 km downstream site. Interestingly, G6PD activity was reduced at the 8 km downstream site, perhaps indicating a shift from the production of NADPH to the production of NADH in mussels exposed to urban effluents in support of β-oxidation during lipid synthesis (Mori et al. 1967).

The responses obtained with MT, labile zinc and COX activity suggest that MT induction might not be related to metal induction in mussels exposed to mixed contaminants from a municipal wastewater source. Indeed, labile zinc levels in gonad and gills were significantly reduced, with a concomitant rise in COX activity. In a previous study at the same study sites, metal accumulation was also lower at the two downstream sites than at the upstream ones, even though MT was induced (Gagnon et al. 2006). Moreover, the investigators found very few meaningful relationships between metal exposure and mussel response (i.e. MT levels), suggesting that low- and high-molecular-weight dissolved organic matter influenced metal availability in gills. Only total copper was significantly elevated in gonad tissues, although labile zinc levels were significantly reduced. MT was implicated in the mobilization of Zn in tissues (Hamilton and Merhle, 1986). In fact, Zn bound to MT was considered as a labile Zn form albeit much less than the dissolved Zn^2+^. The proposed concept behind the labile Zn biomarker was that when metals enter cells a displacement of essential metals such as Zn or copper occurs. Hence the proportion of the labile form of essential metals are increased under exposure to divalent heavy metals and this was proposed as another biomarker of the early biological effects of divalent metals (Gagné and Blaise, 1996). Exposure of zinc led to a significant increase in MT concentrations in *C. fluminea* whereas no significant changes in MT were observed in zebra mussels (Marie et al. 2006). These studies indicate that the rise in Zn in tissues is not always associated in increased gene expression for MT suggesting that the concomitant measurement of labile zinc and MT in tissues was of value to determine exposure and early biological effects to heavy metals.

The estrogenic potential of municipal effluents appears not to contribute to the increase in MT and concomitant decrease in labile zinc pools in tissues. In estradiol-treated trout, hepatic MT remains at basal levels until the Vg mRNA levels begin to decline and Zn is released from high-molecular-weight proteins (Olsson and Kling, 1995), because vitellogenin is a zinc-containing protein in *Xenopus leavis* (Montorzi et al. 1995) and in *Mya arenaria* clams (Gagné et al. 2002). In the present study, zinc levels in the gonad appeared to be negatively related to vitellogenin-like proteins which are egg yolk proteins whose synthesis are under the influence of estrogens, in part at least (Gagné et al. 2001). A decrease in zinc in synaptic vesicles caused by estrogens has been reported in mouse brain (Lee et al. 2004). MT levels were reduced during zinc deficiency, but its association with MT was shown to have protective effects in mice (Kelly et al. 1996).

The increase in MT levels and concomitant decrease in the accumulation of various heavy metals (Gagnon et al. 2006) and labile zinc in gonad and gill tissues might be explained by the inflammation hypothesis. Municipal effluents were recently identified as serotonergic and inducers of COX activity in *Elliptio complanata* mussels (Gagné et al. 2006). MT was reported to be induced by other stressors such as heat stress and during bacterial infection/inflammation (Regala and Rice, 2004). In striped bass and channel catfish, MT expression rose within 24 h in phagocytes as a result of exposure to mycobacterium, suggesting that care should be taken to distinguish between inflammation-induced and metal-induced MT when using MT gene/protein expression as a biomarker of metal exposure. The effluent underwent physical and chemical treatment without any disinfection treatment thereby containing very high amounts of micro-organisms. In a study on rat hepatocytes, the free zinc measured in cells was shown another means for tracking MT in cells when induced by zinc (Coyle et al. 1994). Interestingly, MT was increased by interleukin-1α (a mediator of inflammation) and dexamethasone, but labile zinc levels remained unchanged in rat hepatocytes, suggesting the use of labile zinc as a complementary tool to confirm MT induction by heavy metal exposure (metals would induce both MT and labile zinc in tissues while other non-metallic agents would induce only MT). In another study, MT was induced by glucocorticoids such as cortisol in primary cultures of rainbow trout hepatocytes (Hyllner et al. 1989). In the light of the data obtained, the MT biomarker alone was inadequate for detecting the early biological effects of heavy metals in aquatic organisms exposed to a complex mixture of contaminants like urban wastewater. The measurement of inflammation (i.e. COX activity) and labile zinc levels in tissues was useful in ascertaining the etiological factors of MT induction in various tissues in bivalves. In fact, the analysis of MT levels should be examined against inflammation and labile zinc or other confounding effects using an analysis of covariance model for field studies. For example, an analysis of covariance with gill MT as the variable and COX activity as the covariate revealed that MT was not significantly different from the upstream to the downstream sites and that COX activity had a greater effect on MT levels than did labile zinc or the metal levels reported in tissues. This further suggests that the MT response was related more to inflammation (COX activity) than to levels of labile zinc in gills or metal bioaccumulation.

Another possible explanation for the increase in MT response in the absence, if not the reduction, of labile zinc levels in tissues may have to do with a counteractive reaction of the organisms to maintain essential metals in tissues. Municipal effluents release high molecular weight compounds or colloids in the nanoscale range like humic and fulvic acids that could account for up to 50% of the dissolved organic carbon (Imai et al. 2002). These compounds could act as metal scavengers and dampen the levels of available essential metals in tissues. Hence, MT would be induced to keep zinc and copper homeostatis in cells. However, this hypothesis, wherein MT responds to metal-reactive colloidal organic carbon or other macromolecules to protect against the loss of essential metals, needs more in-depth, specific examination. This hypothesis was supported by the observation that the acid-reactive form of metals was readily increased for essential metals such as zinc and copper, with a concomitant decrease in the truly dissolved fraction for zinc in the municipal plume (Gagnon et al. 2006).

In conclusion, changes in the levels of MT in the gills and gonad exposed to complex mixtures of metals, organic compounds and microorganisms like municipal wastewaters seems not related to heavy metal exposure, but rather to either inflammation or metal scavenging processes in the municipal plume. The finding that mussels at the downstream site accumulated less metals and less labile zinc in their tissues with increased MT and COX expression supported this hypothesis. Exposure to heavy metals and stress should be assessed by a multiple biomarker approach to better discriminate the etiological factors that modulate MT expression. This study also revealed that the exposure of freshwater mussels to a primary-treated effluent produces an inflammation response that may account for the MT responses even up to 12 km downstream of the effluent within the dispersion plume.

## Figures and Tables

**Table 1:** Correlation analysis of biomarker data.

Variable 1	Variable 2	R	P value			
			<*0.001*	<*0.01*	<*0.05*	<*0.1*
Lipogenic enzymes						
ICD gonad	COX gonad	0.89	X			
	MT gills	−0.64		X		
	COX gills	−0.49			X	
	Zn digestive gland	0.62			X	
G6PD gonad	Zn gonad	0.54		X		
	COX gonad	−0.45			X	
	MT gonad	−0.46			X	
Metallothioniens						
MT gonad	Zn digestive gland	−0.5		X		
	MT digestive gland	0.39		X		
MT digestive gland	COX gonad	0.51		X		
	MT gonad	0.39		X		
	COX gills	0.4			X	
	MT gills	0.36		X		
MT gills	COX gonad	0.61	X			
	COX gills	0.62	X			
	Zn digestive gland	−0.56	X			
Labile zinc levels						
Zinc gonad	Zn gills	0.56	X			
Zinc gills	See above					
Zinc digestive gland	See above					
COX						
Gonad	See above					
Gills	See above					
